# Comparison of Nutrition and Flavor Characteristics of Five Breeds of Pork in China

**DOI:** 10.3390/foods11172704

**Published:** 2022-09-05

**Authors:** Yin Zhang, Yingjie Zhang, Hui Li, Tianrong Guo, Jianlin Jia, Pengcheng Zhang, Linguo Wang, Ning Xia, Qin Qian, Haichuan Peng, Zhongli Pan, Dayu Liu, Liming Zhao

**Affiliations:** 1 Meat Processing Key Laboratory of Sichuan Province, Chengdu University, Chengdu 610106, China; 2 Chengdu Institute of Food Inspection, Chengdu 611130, China; 3 Department of Biological and Agricultural Engineering, University of California, Davis, CA 95616, USA; 4 State Key Laboratory of Bioreactor Engineering, R&D Center of Separation and Extraction Technology in Fermentation Industry, East China University of Science and Technology, Shanghai 200237, China

**Keywords:** pork, nutrition, flavor, taste, EUC value, umami, meaty

## Abstract

To characterize the quality of widely consumed pork in China, the chemical compositions and other indexes of five breeds of pork were compared. The results indicated that the moisture content, sensory flavor, and overall acceptability of Pipa pork (PPP) were superior to other breeds. The fat content and essential amino acids (EAA) of Yihao native pork (YNP) were significantly (*p* < 0.05) higher than in other breeds. The protein content, the total amount of amino acids, and perceptible flavor of Tibetan black pork (TBP) were higher than in other breeds. The protein nutrition profiles of manor black pork (MBP) and TBP were better than in others. The equivalent umami concentration (EUC) value of white pork (WP) was significantly (*p* < 0.05) higher than in others; however, the health risk of its fatty acid content was higher. There were unique protein and flavor chemicals in YNP, TBP, and PPP, which may be useful for distinguishing their authenticity.

## 1. Introduction

Pork is a widely consumed meat worldwide because of its large output, nutrient-rich nature, ease of cooking, widely accepted flavor, and other superior characteristics [1,2]. According to statistics from the Food and Agriculture Organization of the United Nations (FAO), the global pork output was 109.835 million tons in 2020, accounting for more than one-third of the total meat output. The output amount of China is the largest worldwide (42.102 million tons) [3,4]. Although the pork output of China is the largest, the domestic pork supply still cannot meet the market demand for sustained population growth. To compensate for the market shortage, China has been importing pork in recent years. China imported 4.2 million tons of pork from other countries in 2020 alone [5]. Simultaneously, the cultivation of pig breeds has increased, and many new pig breeds have emerged, such as manor black pigs (MBP), Tibetan black pigs (TBP), Pipa pigs (PPP), Yihao native pigs (YNP), and white pigs (WP) [6]. Currently, these pig breeds are widely consumed pork breeds in China. However, little information is available on the nutrition, processing and eating qualities, taste ingredients, and volatile aroma compounds of these pork breeds. As we all know, the quality characteristics of pork decide its market value and consumer preferences [7]. Therefore, understanding the quality characteristics of these pork breeds is conducive to understanding the demand of Chinese consumers for different breeds of pork and, thus, promoting international pork participation in China’s market competition.

Nutrient content, texture, and flavor are important quality indices for meat and meat products [8,9,10,11,12]. They are frequently adopted to evaluate the quality of raw meat and its processed products and are considered highly correlated with their eating quality [8,9]. Joo, et al. [13] used flavor, taste, and texture as key parameters to assess the addition of soybean-curd residues to the quality of pork patties. Protein, moisture, ash, and other nutrients were used to evaluate the quality of hamburger patties produced with pork leg and head meat [14]. Hoa, et al. [15] analyzed the relationship between pork grade and its fat content, texture, and other quality traits, and found that higher contents of fat and total unsaturated fatty acids contributed to higher pork grade and better eating quality. Ngapo and Vachon [16] used free amino acids and umami-related components to evaluate the quality of pork stored in fresh (4 °C) or chilled (−1.7 °C) conditions and found that the concentrations of free amino acids increased, but nucleotide concentrations decreased with longer aging periods.

Recently, Wojciak, et al. [17] compared the differences in the chemical compositions of organic and conventional pork and found that the contents of protein and some mineral compounds could be considered the key indices to distinguish organic pork cuts from their conventional counterparts. Huang, et al. [18] reported that the purine content is related to tenderness, juiciness, and some sensory qualities of pork. The physicochemical properties and sensory quality of the prepared pork chops were taken as indices to evaluate the effects of L-arginine and L-lysine on the quality attributes of the prepared pork chops. It was found that 0.4 g/100 g L-arginine or 0.4 g/100 g L-lysine could increase the pH, moisture content, and redness of the uncooked pork chops, decrease cooking loss and shear force, and increase the sensory acceptability of cooked pork chops [19]. These investigations indicate that the chemical composition, texture, and flavor are related to the eating quality of pork as well as to the quality evaluation of pork products. Therefore, to characterize the nutrition, processing and eating qualities, taste ingredients, and volatile aroma compounds in the widely consumed pork in China, the chemical composition, nutritional value, texture, and flavor of pork from MBP, TBP, PPP, YNP, and WP were compared.

## 2. Materials and Methods

### 2.1. Raw Pork and Sample Preparation

Pork samples from five different pig breeds (one-year-olds) were compared. Longissimus dorsi muscles were sampled from six pigs of each breed. The five breeds were the Yihao native pig (YH, binary hybrid of Luchuan × Taihu pig, 80–85 kg) bought from Guangdong Yihao Foodstuff Co., Ltd., Zhanjiang, China; Tibetan black pig (TB, quaternary hybrid of the Meishan pig × Huchuan mountain pig × Tibetan pig × Buckshire pig, 70–75 kg) bought from Tie Qi Li Shi group, Mianyang, China; manor black pig (MB, binary hybrid of the Tibetan Pig and Sichuan black pig, 75–85 kg) bought from Sichuan GAOJIN Food Co., Ltd., Suining, China; Pipa pig, 50–55 kg bought from Chengdu Xiwang Food Co., Ltd., Xinjing, China; White pig (WP, ternary hybrid of the Meishan pig × Chenghua pig × Landrace hybrid, 70–80 kg) bought from Chengdu Xiwang Food Co., Ltd. All longissimus dorsi muscles were obtained from the slaughterhouse of each company and transported to our laboratory within 24 h after post mortem, shaved, the fascia and fat were removed, and they were refrigerated at 4 °C. To ensure the consistency of samples, the muscle samples were chopped and frozen in liquid nitrogen for 10 min, and then an ultra-micro pulverizer (IKA company, Staufen, Germany) was used to crush them into powder when the chemical compositions of muscle samples were determined.

### 2.2. Chemicals

Nucleotide standard: disodium guanosine 5′-monophosphate (5′-GMP, with purity 99.0%), disodium cytidine 5′-monophosphate (5′-CMP, with purity 99.0%), disodium uridine 5′-monophosphate (5′-UMP, with purity 99.0%), disodium inosine 5′-monophosphate (5′-Imp, with purity 99.0%), disodium adenosine 5′-monophosphate (5′-AMP, with purity 99.0%), and disodium xanthin 5′-monophosphate (5′-XMP, with purity 98.0%) were bought from Sigma company; 16 kinds of standard amino acids, methanol, acetonitrile (special for chromatography) were purchased from Thermo Fisher Technology Co., Ltd., Waltham, MA, USA; concentrated hydrochloric acid (HCl), trichloroacetic acid, sodium hydroxide, sulfosalicylic acid, potassium dihydrogen phosphate, arc bis (polyacrylamide), 10% APS (ammonium persulfate), TEMED (tetramethylethylenediamine), Tris base (trimethylolaminomethane), glycine, SDS (sodium dodecyl sulfate), 1m Tris HCl (trimethylolaminomethane hydrochloride, pH 6.8), glycerol, 2-mercaptoethanol, bromophenol blue, Brilliant Blue R and other chemicals were of analytical grade and bought from Chengdu Cologne Chemicals Co., Ltd., Chengdu, China.

### 2.3. Determination of Chemical Compositions

Moisture, protein, fat, fat acids, and ash content of the meat samples were analyzed according to the national food safety standard of China. The moisture content was determined by the direct drying method at 103 ± 1 °C with a PCD-E3000 constant temperature blast drying oven (Shanghai Langgan Experimental Equipment Co., Ltd., Shanghai, China) [20]. The protein content was analyzed by the Kjeldahl method [21]. The fat content was determined by the Soxhlet extraction method [22]. The fatty acid content was determined with a 7890A Agilent gas chromatograph equipped with column DB-FastFAME (90 m × 0.25 mm × 0.25 µm) after hydrolysis extraction of the meat samples [23]. The ash content was determined by the high-temperature burning method [24]. Triplicate determinations were performed using three samples for each muscle sample.

### 2.4. Determination of Fatty Acid Profile

The fatty acid profiles of the meat samples were determined according to the national food safety standards of China GB 5009.168-2016 [23]. Briefly, 3 g of a pork sample was mixed with 1.0 mL of tridecanoic acid (10 mg/mL), 100 mg of pyrogallic acid, several zeolites, 2 mL of 95% ethanol, 4 mL of water, and 10 mL of 8.3 mol/l HCl. The flask was heated and shaken in a water bath at 75 °C for 40 min to hydrolyze the fat. After hydrolysis, we added 10 mL of 95% ethanol into the cooled flask (26–27 °C), transferred it to a separating funnel after mixing, rinsed the flask with 50 mL of ether and petroleum ether mixture (*v*:*v* = 1:1), put the rinsing solution into the separating funnel, shook it for 5 min, and let it stand for 10 min. A rotary evaporator was used to concentrate the hydrolysate to obtain the fat extract. We added 8 mL of 2% potassium hydroxide methanol solution to the extract and then refluxed it at 80 °C in a water bath until all the fat extract was dissolved. We added 7 mL of 15% boron trifluoride methanol solution into the fat extract mixture, placed it into the water bath at 80 °C for 2 min, added 2.0 mL of saturated sodium chloride solution and 5.0 mL of n-hexane solution into the fat extract mixture, and shook well. After standing, the supernatant was sampled and the fatty acid profiles were analyzed with an Agilent 7890A (Agilent company, Santa Clara, USA) gas chromatograph with an Agilent DB-FastFAME (90 m × 0.25 mm × 0.25 µm) column and an FID detector; hydrogen was as a carrier gas. The tridecanoic acid methyl ester (FAME) was used as the reference standard for the analyses. The temperature program for the fatty acid determination was 75 °C for 1 min followed by a temperature increase of 35 °C/min to 200 °C, held at this temperature for 14 min; followed by an increase of 2.5 °C/min to 210 °C, and run at this temperature for 5 min, and then followed by an increase of 12 °C/min to 230 °C, and held at this temperature for 20 min until all the fatty acids were detected. The results were calculated as mg of fatty acids per 100 g of meat. Based on the fatty acid profile, the hypocholesterolemic/hypercholesterolemic (h/H) [25] ratio, the nutritional value (NV) [26], the indices of atherogenicity (IA) [27], and thrombogenicity (IT) [27] were compared. The calculation formulas were as follows:

$$\mathrm{IA}=\frac{\left(C12:0+4\times C14:0+C16:0\right)}{\mathrm{MUFA}+\text{n-6}\;\mathrm{PUFA}+\text{n-3}\;\mathrm{PUFA}}$$

$$\mathrm{IT}=\frac{\left(C14:0+C16:0+C18:0\right)}{(0.5\times \mathrm{MUFA}+0.5\times \text{n-6}\;\mathrm{PUFA}+3\times \text{n-3}\;\mathrm{PUFA}+\frac{n-3}{n-6})}$$

$$\mathrm{NV}=\frac{\left(C12:0+C14:0+C16:0\right)}{\left(C18:1\;\text{n-9}+C18:2\;\text{n-6}\right)}$$

$$\frac{h}{H}=\frac{C18:1+C18:2\;\text{n-6}+C18:3\;\text{n-3}+C20:3\;\text{n-6}+C20:4\;\text{n-6}+C20:5\;\text{n-3}+C22:5\;\text{n-3}}{C14:0+C16:0}$$

### 2.5. Determination of Free and Hydrolyzed Amino Acids

Free and hydrolyzed amino acids were determined. The free amino acids were determined according to Zhang, Li, Zhang, Wang, Zhang, Jia, Peng, Qian, Zhang, Pan, Liu and Zhao [4], and Zhang, et al. [28], with some modifications. A total of 100 mg of muscle sample was blended with 50 mL 0.1 mol/L HCl and treated for 30 min using a KQ3200DB ultrasonic oscillator at room temperature (26–27 °C) (Kunshan Ultrasonic Instrument Corporation, Ltd., KunShan, China). The mixture was centrifugally separated at 1744 g for 10 min. The obtained supernatant was shifted to a 100 mL volumetric flask. The residue material was extracted twice and we mixed the supernatant after centrifugal separation. The supernatant was mixed with 5% 5-sulfosalicylic acid and reacted for 5 min. To remove precipitation, a qualitative filter paper (Fushun Mingzheng filter paper factory, Anhui, China) was used to filter the mixture. A sodium hydroxide (NaOH) solution was used to adjust the pH of the supernatant to 2.2.

The hydrolyzed amino acids were analyzed according to the method of Sun, et al. [29], with some modifications. The crushed muscle (100 mg) was sealed after filling with nitrogen gas and hydrolyzed it using 10 mL 6 mol/L HCl in a constant temperature drying oven at 110 ± 1 °C for 24 h. The mixture was cooled and a glass filter was used to filter it. To completely remove the solvent, the filtrate was put into a rotary vacuum evaporator (Zhengzhou Kaixiang Instrument Equipment Co., Ltd, Zhengzhou, China) and concentrated at 58 °C. Sodium citrate buffer was used to adjust the amino acid concentration to 50–250 nmol /mL.

The solution was passed through a 0.45 µm pore size microfiltration membrane (Shanghai Xinya Purification Equipment Co., Ltd., Shanghai, China). Phthalic dicarboxaldehyde was used to derive the filtrate. After precolumn derivatizing, the filtrate was subjected to a reversed-phase high-performance liquid chromatography (RP-HPLC) SYKAM Amino Acid Analyzer S 433D (Sykam GmbH, Kleinostheim, Munich, Germany) equipped with a PEEK column (4.6 × 150 mm, 7 µm, 10% cross-link) to determine the amino acids. Triplicate determinations were performed, and the mean values were calculated.

### 2.6. Protein Nutrition Evaluation

Based on the amino acid contents of the muscles, the protein nutrients of the five pig breeds were evaluated according to Sun, Yue, Zhang, Li and Zhang [29] with some modifications. The protein values of muscles were analyzed by six parameters; they were amino acid score (AAS), essential amino acid index (EAAI) [30], and protein efficiency ratio [31]. The calculation formula of those parameters is as follows:

$$\mathrm{AAS}=\mathrm{Min}\left\{{\left(\mathrm{aa}/\mathrm{AA}\right)}_{1},\ldots ,{\left(\mathrm{aa}/\mathrm{AA}\right)}_{k}\right\}$$

$$EAAI=\sqrt[{n}]{\prod_{i-1}^{n}\left(\frac{A_{i}}{B_{i}}\times 100\right)}$$

PER =−0.468+0.454(Leu)−0.105(Tyr)

where *Ai* is the essential amino acid in the pork sample, mg/g; n—the number of amino acids; m—the number of essential amino acids; *Bi*—the amino acid content in the reference pattern of the protein requirement [30]; aa—the amino acid content of the pork sample, AA—the amino acid content of the suggested pattern of the protein requirement [30], k—the number of amino acid types [30], Leu—leucine, Tyr—tyrosine.

### 2.7. Texture Profile

The texture profile (hardness, adhesiveness, gumminess, springiness, chewiness, cohesiveness, and resilience) was measured by a TA. The Xt plus texture analyzer (Stable Micro Systems, Godalming, Surrey, UK) was at room temperature (26–27 °C). Prior to analysis, muscle samples (5 cm × 5 cm × 2 cm) were put into a cooking bag and heated in an HH-6 thermostatic water bath (Changzhou Aohua Instrument Corporation, Ltd., Changzhou, China) at 96 °C for 20 min. The muscle samples were equilibrated to room temperature for 1.5 h and trimmed into 2 cm × 2 cm × 1.5 cm pieces. Six trimmed muscle pieces of each pig breed were put on the sample table. The muscle sample was compressed with a cylindrical P45 probe at a constant speed of 1 mm/s. The muscle was pressed in two consecutive cycles of 50% compression with 5 s between cycles.

### 2.8. Cooking Loss

The cooking losses of the five pig breeds were evaluated according to Honikel [32]. Pork piece samples (20 g of each pork piece) from each pig breed were put into a cooking bag and heated in an HH-6 thermostatic water bath (Changzhou Aohua Instrument Corporation, Ltd., Changzhou, China) at 80 °C. As the center temperatures of meat pieces reached 70 °C, the meat pieces were kept at 70 °C for 30 min, and then were taken out and left to stand for 20 min to cool naturally to room temperature (26–27 °C). The visible exudates on the surfaces of the meat pieces were manually removed with filter paper. The cooking loss was calculated as the percentage of the weight loss based on the difference between the sample weights before and after heating. Three repetitions were performed and the mean values were reported.

### 2.9. Color Measurements

The color of each pork piece was measured with a CR-400 colorimeter (Konica Minolta Sensing, Inc., Sakai Osaka, Japan). The view angle and illumination area of the colorimeter were 2° and φ 8 mm, respectively. The colorimeter was calibrated using a white calibration plate and the parameters L *, a *, and b * (CIELAB scale) were recorded. Ten readings were performed on the surface of each sample. The results were expressed as L *, a *, b *. Seven determinations were carried out and the mean values were reported.

### 2.10. Sensory Evaluation

Pork pieces (5 cm × 5 cm × 2 cm) of every pig breed were put into a cooking bag and heated in an HH-6 thermostatic water bath (Changzhou Aohua Instrument Corporation, Ltd., Changzhou, China) at 96 °C for 20 min. After cooking, the muscle samples were trimmed into 2 cm × 2 cm × 1.5 cm pieces immediately. When the pork pieces were warm (60 ± 5 °C), 6 pieces of every pig breed were served for panels to perform a sensory evaluation. Quantitative descriptive analysis [33] was carried out to assess the flavor, meaty (umami), sensory springiness, and appearance of pork pieces. The panel consisted of 12 trained assessors [34,35]. The generation and selection of scoring criteria were carried out by open discussions in six sessions prior to the experiments. The scoring criteria retained for the sensory evaluation are described in Table 1. A non-structured scoring scale was used, where 0 and 20 meant the lowest and the highest, respectively. The sensory analysis was carried out in five sessions for each kind of pork piece using a complete block design, where each panelist evaluated one kind of pork piece from each test (three times in each session). Triplicate sensory evaluations were performed for each kind of pork.

### 2.11. SDS–Polyacrylamide Gel Electrophoresis (SDS–PAGE)

The SDS–PAGE analysis was used to compare the protein patterns according to the methods of Zhang, et al. [36] and Liu, et al. [37]. A total of 20 mL of 0.5 mol/L Tris–HCl (pH 7.8) was mixed with 2 g of crushed pork muscle powder. The mixture was centrifugally separated at 7000 g for 10 min. The solubilized sample was dissolved at 4:1 (*v*/*v*) ratio with the buffer solution (pH 6.8, 1 mol/L Tris–HCl, containing SDS [2 g/100 mL], glycerol [25 mL/100 mL], bromophenol blue (0.1 g/100 mL), and β-ME (5 mL/100 mL)). The pork samples (20 mg protein) were loaded into the polyacrylamide gel made with 10% running gel and 5% stacking gel and were electrophoretically separated at a constant current of 15 mA. After electrophoresis, the proteins were stained with Brilliant Blue R (0.1 g/100 mL) in methanol (45 mL/100 mL) and acetic acid (10 mL/100 mL), and de-stained with the mixture of methanol (10 mL/100 mL) and acetic acid (10 mL/100 mL). A Bio-Rad imaging scanning densitometer (Versa Doc 3000, Bio-Rad, Milan, Italy) was used to scan the electrophoresis gel. The molecular weight of the protein in SDS–PAGE was calculated with the method of Zhang, et al. [38].

### 2.12. Determination of 5‘-Nucleotides

The determination of 5‘-nucleotides in pork was according to Poojary, et al. [39] and Zhang, Li, Zhang, Wang, Zhang, Jia, Peng, Qian, Zhang, Pan, Liu and Zhao [4], with some modifications. The 5′-mononucleotide analysis was determined with an Agilent 1100 HPLC (Agilent company, USA) equipped with a UV detector and Hypersil ODS2-C18 (4.6 mm × 250 mm, 5.0 μm). The weighed 20 g of crushed muscle powder was used. An external standard method was used to perform quantitative determination and with parallel determination (three times). All determinations were conducted in triplicates. The 5′-mononucleotide contents were expressed as mg of 5′-mononucleotide per 100 g of pork muscle (mg/100 g).

### 2.13. Equivalent Umami Calculation

The equivalent umami calculation (EUC) was performed according to Yamaguchi, et al. [40] and Zhang, Li, Zhang, Wang, Zhang, Jia, Peng, Qian, Zhang, Pan, Liu and Zhao [4]. The EUC equation is:

$$\mathrm{EUC}\;=\sum a_{i}b_{i}+1218(\sum a_{i}b_{i})(\sum a_{j}b_{j})$$

where EUC represents the umami intensity given by 1 g of MSG (g MSG/100 g), a_i_ represents the concentration (g/100 g) of umami amino acid Glu and Asp; a_j_ represents the concentration (g/100 g) of each umami 5′-mononucleotide (IMP, GMP, AMP, and XMP); b_i_ represents the relative umami concentration for each umami amino acid to MSG (Asp = 0.077; Glu = 1); b_j_ represents the relative umami concentration for the umami nucleotide to IMP (IMP = 1; XMP = 0.61; GMP = 2.3; AMP = 0.18); and 1218 is a synergistic constant based on the concentration (g/100 g) used.

### 2.14. Analysis of Volatile Aroma Compounds

The volatile aroma compounds of the crushed pork (2.1) were measured according to Zhang, Zhang, Venkitasamy, Guo, Pan, Ke, Tang, Huang and Zhao [11], Chen, et al. [41], and Zhang, Li, Zhang, Wang, Zhang, Jia, Peng, Qian, Zhang, Pan, Liu and Zhao [4]. A 7890B-5977A gas chromatography–mass spectrometer (Agilent Technologies, Inc., Santa Clara, CA, USA) with a CTC automatic sampler was used to quantify and identify the volatile aroma compounds in the pork. The crushed pork was put into a sample bottle and loaded onto a CTC automatic sampler to analyze the volatile aroma compounds. A HP-5MS UI-type capillary column (coating 0.25 µm, inner diameter 0.25 mm, length 30 m; Agilent, Santa Clara, CA, USA) was adopted for GC separation. The heating time of the CTC automatic sampler was 30 min and the heating temperature was set at 60 °C. The analysis time was 5 min. Helium gas was used as the carrier gas and flowed at a constant speed of 1.0 mL/min. The temperature program for the volatile aroma compounds determination was 1.0 min at 70 °C, a ramp of 3 °C/min to 85 °C, then increased to 105 °C at 3 °C/min and stood for 2 min, then increased to 165 °C at 12 °C/min and stood for 2 min, and then increased to 230 °C at 8 °C/min and stood for 5 min. The mass spectrometer was run in the electron impact mode at 70 eV (ion source temperature: 230 °C). A full scan mode (m/z 33–400) was used to record the samples, the emission current was 100 μA, and the detection voltage was 350 V. The 2, 4, 6-trimethylpyridine with a concentration of 0.01 mol/L was used as the internal standard. Identification of the volatile aroma compounds in the pork sample was achieved by comparing its retention times and mass spectra to the NIST14.0 mass spectrum library; the compound’s compatibility degree larger than 80% was selected. The formula for calculating the absolute content of unknown compounds [17] and the odorant activity value (OAV) is as follows:

$$C_{i}=\frac{\rho \times V\times A_{i}}{A\times m},\;OVA=\frac{C_{i}}{OT_{i}}$$

where *C_i_* is the absolute content of the unknown compound, μg/kg; *ρ* is the mass concentration of the internal standard (2 μg/μL); *V* is the volume of the internal standard; *A* is the peak area of the internal standard substance; *A_i_* is the peak area of each component; m is the mass of the sample (kg); *OAV* is the odorant activity value; *OT_i_* is the compound threshold.

### 2.15. Statistical Analysis

The results were analyzed by ANOVA at a significance level of 5% (H0: *p* < 0.05), *p* ≥ 0.05 was defined as a non-significant difference. All of the tests were conducted in triplicates. The comparison of means was analyzed by Fisher’s LSD tests using the SAS 9.0 statistical package.

## 3. Results and Discussion

### 3.1. Pork Nutrition

#### 3.1.1. Proximate Composition

Proteins, fats, and minerals are important nutrients in pork and are of great importance to human health [7]. To compare the basic nutrients in the five breeds of pork, their chemical compositions were determined (Table 2). The water, protein, and fat contents of the five pork samples were significantly different (*p* < 0.05), whereas the ash content of the five pork samples was not significantly different (*p* ≥0.05). The moisture content of PPP, fat content of YNP, and protein content of TBP were the highest compared to the other pork samples.

Compared to other nutrients, the fat content of pork was considered more important and was considered a key index to define the overall carcass value. A possible explanation for this was that fat greatly influenced the flavor of pork and its processed products [8] and was correlated with its quality grade; the higher grade of pork had a higher fat content [15]. To further compare the nutritional value of protein and fat, fatty acids and protein nutrition were evaluated.

#### 3.1.2. Fatty Acid Profile

The fatty acids in the five breeds of pork are shown in Table 3. There were 16, 14, 14, 13, and 11 types of fatty acids in YNP, MBP, PPP, TBP, and WP, respectively. C16:0 (palmitic acid, 24.13–28.07%), C18:0 (stearine acid, 12.19–13.36%), C18:1n9 (oleic acid, 35.54–45.30%), and C18:2n6 (linoleic acid, 7.20–17.58%) were the main fatty acids of the five breeds of pork, and their contents were higher than those of other fatty acids. These phenomena were consistent with the results of Choi, et al. [42]. Compared to the other four breeds of pork, the content of polyunsaturated fatty acids (PUFA) of TBP was the highest, and the contents of saturated fatty acids (SFA) and monounsaturated fatty acids (MUFA) of PPP were higher than in the other pork samples.

It has been reported that the PUFA content (C18:2n6, C18:3n3, C20:3n6, and C20:4n6) was negatively correlated with pork flavor and overall acceptability, whereas C18:1n9 was positively correlated with pork flavor and overall acceptability [43]. The C18:2n6, C18:3n3, C20:3n6, and C20:4n6 contents of PPP were lower than those of the other pork samples, and the C18:1n9 content of PPP was higher than that of the other pork samples. These results suggest that the flavor and overall acceptability of PPP are superior to those of the other breeds of pork. Genetics, hybrids, and feeding conditions may explain the difference in fatty acids among the five breeds of pork [44].

The health risk of eating pork fat has been a major concern in recent years and is considered a cause of diseases, including cancer, cardiovascular disease, and diabetes [45]. To evaluate the health influences of the five breeds of pork, the index of thrombogenicity (IT), index of atherogenicity (IA), hypocholesterolemic/hypercholesterolemic ratio (h/H), and nutritional value ratio (NV) were calculated according to the value of the fatty acids (Table 3). The IA, NV, and IT of WP were higher than those of the other breeds of pork, the IA and NV of the other breeds of pork were similar, and the IT of PPP and YVP were higher than those of MBP and TBP. The h/H values of MBP and TBP were higher than those of the other three breeds of pork; WP had the lowest h/H value. The Pearson’s correlation analysis of IA, NV, IT, and h/H indicated that there was a high correlation between them; the correlation and correlation coefficients of IA and IT, IA and NV, IA and h/H, IT and NV, IT and h/H, and NV and h/H were 0.989, 0.991, −0.999, 0.997, −0.985, and −0.986, respectively. Therefore, the higher IA, NV, and IT of WP suggest that the health risk of eating WP fatty acids is higher than that of the other breeds of pork.

#### 3.1.3. Protein Nutrition

##### Protein Expression Patterns

**Figure 1:**
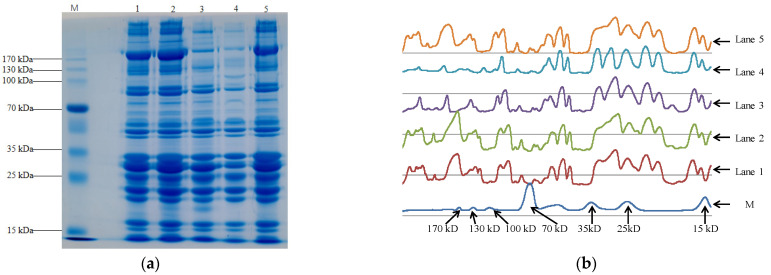
Sodium dodecyl sulfate–polyacrylamide gel electrophoresis (SDS–PAGE) and optical density of pork muscle protein. (**a**) is SDS–PAGE image of the five breeds of pork; (**b**) is the optical density (O.D.) of SDS-PAGE image; MBP, manor black pigs; TBP, Tibetan black pigs; PPP, Pipa pigs; YNP, Yihao native pigs; WP, white pigs; M, marker; lanes 1, 2, 3, 4, and 5 are the protein expression patterns of the muscles from MBP, TBP, PPP, YNP, and WP, respectively.

The protein expression patterns of the five breeds of pork are shown in Figure 1. The optical density (O.D.) of sodium dodecyl sulfate–polyacrylamide gel electrophoresis (SDS–PAGE) (Figure 1b) was analyzed to study protein expression patterns. The results indicated that the protein expression patterns of the five breeds of pork were similar at molecular weights <130 kDa; but different at molecular weights >130 kDa. PPP and YNP had lower molecular weights (>130 kDa) than the other breeds of pork. The protein expression pattern of YPN was different from that of the other samples; there was a unique protein with a molecular weight of 113.257 kDa; however, it lacked a protein with a molecular weight of 124.368 kDa compared to the other breeds of pork. Both TBP and PPP had a unique protein with a molecular weight of 15.948 kDa compared to the other three breeds of pork. The O.D. of MBP and TBP was higher than that of the other breeds of pork, with higher O.D. indicating higher protein content. This result was consistent with the protein content of MBP and TBP being higher than that of the other breeds of pork (Table 2).

SDS–PAGE is widely used to distinguish and identify the category, age, and quality of edible meat [46,47,48]. The difference in protein molecular weights >130 kDa, the unique protein with a molecular weight of 113.257 kDa in TPN, and the protein with a molecular weight of 15.948 kDa in TBP and PPP indicated that the proteins in the five breeds of pork were different from each other. Pork genes determine the type, structure, and function of pork proteins [49]. Therefore, the difference in pork genes may explain the difference in the proteins of the five breeds of pork. The unique proteins identified in this study may be useful for the identification of the pork category and characterization of its function.

##### Amino Acid Composition and Nutrition

To evaluate the protein nutrition of the five breeds of pork, the amino acid composition, and nutritional value indices were determined (Table 4). The amino acid contents of the five breeds of pork were significantly different (*p* < 0.05), and the total amount of amino acids in TBP was the highest (significantly higher (*p* < 0.05) than that in the other breeds of pork). This was consistent with the highest protein content of TBP (Table 2). The total essential amino acid(s) (EAA) of the five breeds of pork were similar; however, the EAA of YNP was the highest.

According to the recommended protein model of the FAO/World Health Organization (WHO), the protein nutrition indices of the amino acid score (AAS) and essential amino acid index (EAAI) for children and adults were calculated (Table 2). The data indicated that the protein nutrition indices of AAS and EAAI for children and adults were different from those of the five breeds of pork. According to the protein models for children and adults, the first limiting amino acids of the five breeds of pork were threonine (Thr) and histidine (His), respectively. For MBP, the AAS for adults and EAAI for children and adults were the highest; however, the AAS for children of MBP was lower than that of WP and higher than that of the other breeds of pork. The AAS and EAAI for adults and children of TBP were the second highest. Given that EAAI represents all the amino acids of the protein model, the AAS was calculated using one amino acid of the protein model [30], the EAAI is more representative than the AAS in characterizing protein nutrition [29]. Therefore, the EAAI values in Table 4 suggest that the protein nutrition of MBP and TBP was higher than that of the other breeds of pork.

### 3.2. Pork Processing and Eating Qualities

#### 3.2.1. Texture Profile

Meat texture is important to its eating quality and has been used as a key index for evaluating meat quality [50,51]. The texture profiles of the five breeds of pork (Table 5) indicated that the hardness of MBP was significantly (*p* < 0.05) higher than that of the other breeds of pork, and the hardness of YNP, WP, TBP, and WP were not significantly different (*p* ≥ 0.05). However, the hardness of YNP was higher than that of WP and TBP, and the hardness of PPP was the lowest. The gumminess of the five breeds of pork exhibited similar differences as the hardness. The adhesiveness of PPP and MBP was significantly higher (*p* < 0.05) than that of the other pork breeds. The cohesiveness and resilience of MBP, PPP, and YNP were significantly (*p* < 0.05) higher than those of the other pork breeds. The chewiness of MBP and YNP and the springiness of PPP were significantly higher (*p* < 0.05) than those of the other pork breeds. The results of Pearson’s correlation analysis indicated that there was a significant correlation between hardness and gumminess (r = 0.985, *p* < 0.01), springiness and adhesiveness (r = 0.914, *p* < 0.05), springiness and cohesiveness (r = 0.922, *p* < 0.05), and cohesiveness and adhesiveness (r = 0.953, *p* < 0.05).

The meat texture profile is influenced by the breed and age of pigs [52]. The hardness of pork muscle is positively correlated with muscle fiber type IIb and negatively correlated with muscle fiber type IIa [53]. The five breeds of pork were obtained from different pig breeds. The muscle proteins were different (Figure 1). These factors may explain the difference in the texture profiles of the five breeds of pork. The difference in the hardness of the five breeds of pork further supported that the pork was from different breeds. Adhesiveness, springiness, and cohesiveness are three parameters that reflect the internal structural strengths of muscle fibers [54]. The adhesiveness, springiness, and cohesiveness of PPP and YNP were higher than those of the other breeds of pork. This difference may suggest that the muscle proteins of PPP and YNP were not easily dissolved, resulting in their O.D. (Figure 1) being lower than that of the other breeds of pork muscles.

#### 3.2.2. Color

**Figure 2:**
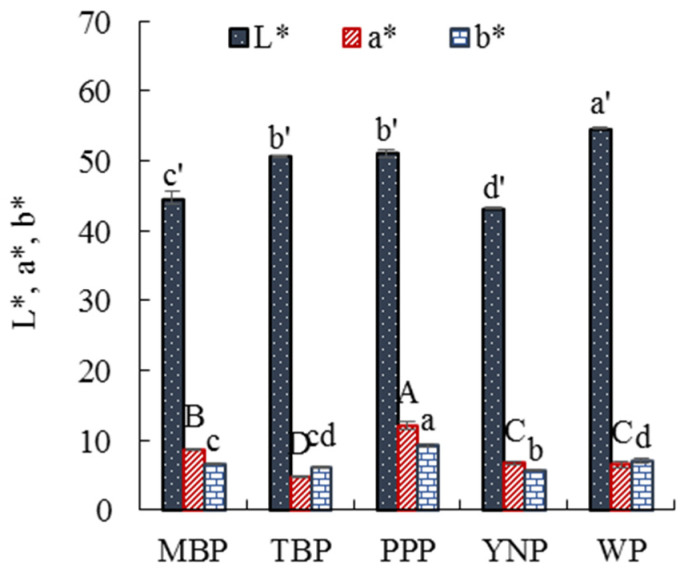
L *, a *, and b * of the five types of pork. Values are shown as the mean ± standard deviation. Different letters above the bars of standard deviations indicate significant differences (*p* < 0.05); MBP, manor black pigs; TBP, Tibetan black pigs; PPP, Pipa pigs; YNP, Yihao native pigs; and WP, white pigs.

Meat color is the first impression of consumers, and the redness of pork is closely related to the purchase time of consumers [55]. The colors of the five breeds of pork (Figure 2) indicated that the a * value of PPP was significantly higher (*p* < 0.05) than that of the other breeds of pork, and the a * value of MBP was significantly lower (*p* < 0.05) than that of PPP but significantly (*p* < 0.05) higher than those of YNP and WP, whose a * values were not significantly different (*p* ≥ 0.05). The a * value of TBP was significantly lower (*p* < 0.05) than that of the other breeds of pork. The L * value of WP was significantly higher (*p* < 0.05) than that of the other pork samples. The L * values of TBP and PPP were not significantly different (*p* ≥ 0.05); however, their L * values were significantly higher (*p* < 0.05) than those of MBP and YNP. The L * value of YNP was the lowest. The b * value of PPP was significantly higher (*p* < 0.05) than that of the other breeds of pork. The b * value of YPN was significantly lower (*p* < 0.05) than that of PPP, but it was significantly higher (*p* < 0.05) than that of MBP and TBP, with b * values not significantly different (*p* ≥ 0.05). The b * value of WP was the lowest.

The results of investigating the relationship between pork color and breed indicated that the L * and a * values of pork were influenced by both hemoglobin content and myoglobin forms, and the b * value was greatly influenced by myoglobin forms in pork [56]. The five breeds of pork were obtained from different pig breeds. The difference in hemoglobin content and myoglobin forms may explain the significant differences in the a * and b * values of the five breeds of pork. A higher intramuscular fat content results in a higher L * value [57,58]. This can be confirmed by the significant correlation (R^2^ = 0.8035) between the L * value and the fat content of the five breeds of pork.

#### 3.2.3. Cooking Loss

**Figure 3:**
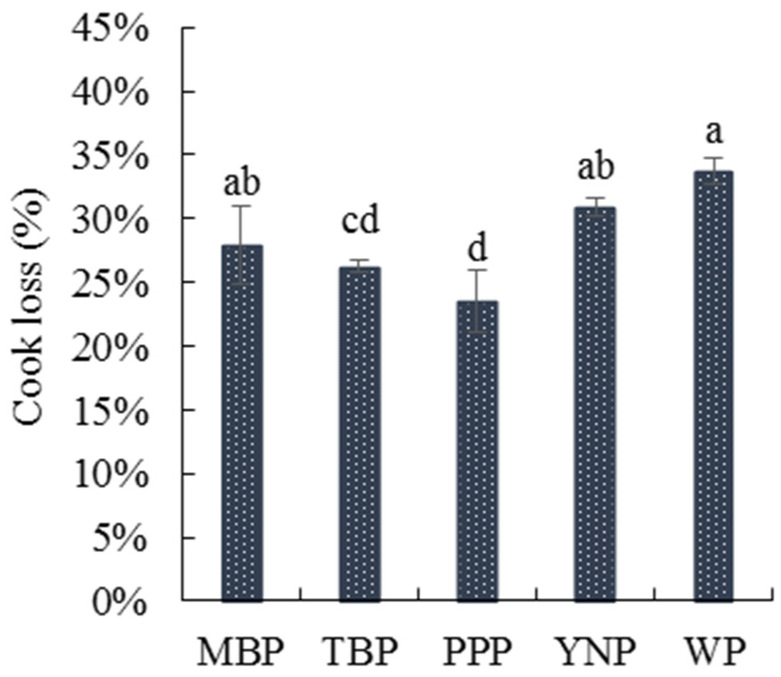
The cooking losses of the five types of pork. Values are shown as the mean ± standard deviation. Different letters above the bars of standard deviations indicate significant differences (*p* < 0.05); MBP, manor black pigs; TBP, Tibetan black pigs; PPP, Pipa pigs; YNP, Yihao native pigs; WP, white pigs.

Cooking loss is a key index for the commercial use of pork; it relates to eating quality, weight loss, and the cost of producing pork [59]. The data in Figure 3 indicated that the cooking loss of the five breeds of pork ranged from 20.06% to 33.75%; the cooking losses of MBP, YNP, and WP were not significantly different (*p* ≥ 0.05); however, their cooking losses were significantly higher (*p* < 0.05) than those of TBP and PPP.

The genome sequence [60], slaughter stress [61], and feeding conditions [62] are influencing factors in the cooking loss of pork. These may be the main explanations for the differences in cooking losses between the five breeds of pork.

#### 3.2.4. Sensory Analysis

To evaluate the eating quality of the five breeds of pork, the pork was boiled, and a sensory evaluation was performed. The results in Table 6 indicate that the flavors of the five breeds of pork were not significantly different (*p* ≥ 0.05); however, the flavor of PPP was the best compared to the other breeds of pork. This was consistent with the volatile aroma compounds of PPP, which was more than others. The meaty taste of PPP was lower than that of the other breeds of pork. This was consistent with the results of the EUC value of PPP, which was the lowest. The springiness of PPP was higher than that of the other pork samples, which was similar to the springiness determined by the instrument (Table 5). The appearance of PPP was better than that of the other pork samples.

The aldehydes were the key aroma compounds that influenced the flavor differences between beef, pork, and fish [63]. The content of aldehydes in PPP was higher than that in the other pork samples (Table 7). This may be an explanation for the better flavor of PPP. The meaty flavor is referred to as the umami taste [12]. The EUC value of PPP was lower than that of the other pork samples. A lower EUC value indicates less umami taste. This may explain why the meaty taste of PPP was the lowest among the five breeds of pork. The PPP from which the sample was obtained was raised on a plateau and the pigs grew slowly [64]. The special feeding environment may have resulted in the springiness of PPP being higher than that of the other breeds of pork. The a * value of PPP was higher than that of the other breeds of pork, which may explain why the appearance of PPP was better than that of the others.

### 3.3. Taste Ingredients

#### 3.3.1. Taste of Free Amino Acids

According to taste differences, amino acids were divided into umami amino acids (Glu, Asp), sweet amino acids (Thr, Ser, Gly, Ala, Pro), bitter amino acids (Val, Met, Ile, Leu, Phe, His, Arg), and tasteless amino acids [65]. Free amino acids are also precursors of Maillard reactions [66] and influence the flavor of cooked meat. Given the important effect of free amino acids on meat taste, the free amino acids in the five breeds of pork were determined (Table 8). The data indicated that the free amino acids were significantly different (*p* < 0.05). The total content of free amino acids in MBP was significantly higher (*p* < 0.05) than that of TBP or PPP, and the total content of free amino acids in TBP was significantly higher (*p* < 0.05) than that in PPP; however, the total content of free amino acids of both was significantly higher (*p* < 0.05) than that in YNP and WP, with free amino acid contents that were not significantly different (*p* ≥ 0.05). The bitter amino acid content in the five breeds of pork showed the same significant difference as that of the total free amino acid content. The sweet amino acid content in MBP was significantly higher (*p* < 0.05) than that of TBP and TNP, with sweet amino acid contents that were not significantly different (*p* ≥ 0.05) but significantly higher (*p* < 0.05) than that of PPP and WP. The content of sweet amino acids in PPP was significantly lower (*p* < 0.05) than that in WP. The content of umami amino acids in TBP was significantly higher (*p* < 0.05) than that in PPP and WP, and the contents of umami amino acids were not significantly different (*p* ≥ 0.05). The content of umami amino acids in MBP was significantly lower (*p* < 0.05) than that in YNP and WP; however, it was significantly higher (*p* < 0.05) than that in PPP.

The pig breed may explain the difference in the contents of free amino acids in the five breeds of pork [67]. Umami and sweet tastes are widely accepted flavors [12,65,68] and have received more attention in the flavor manipulation of meat products or other foods [10,11]. In particular, the umami taste not only endows a food-favorable taste but also improves the nutritional intake for patients and elderly individuals, protects against duodenal cancer, reduces ingestion of sodium chloride, and decreases consumption of fat [68]. Therefore, to make good use of the taste produced by free amino acids, pork with higher contents of umami or sweet amino acids should be used for slightly processed meat products, such as steamed, boiled, and stewed pork. Pork with a high content of free amino acids should be used for roasted or grilled meat products.

#### 3.3.2. Nucleotides and Equivalent Umami Concentration (EUC) Value

In addition to amino acids, some nucleotides are taste-enhancing ingredients as well as flavor precursors of cooked meat products [69]. The taste-enhancing effects of nucleotides and amino acids are the main causes for the umami taste of mushrooms and meat [65,68]. Guanosine monophosphate (GMP), xanthosine monophosphate (XMP), and inosine monophosphate (IMP) are well-known umami enhancers. They have been successfully used in commercial products (for example, chicken essence and soy sauce). The contents of nucleotides in the five breeds of pork (Table 9) indicated that IMP was the main nucleotide found, the IMP content of YNP was significantly higher (*p* < 0.05) than that of WP, the IMP content of WP was significantly higher (*p* < 0.05) than that of TBP, the IMP content of TBP was significantly higher (*p* < 0.05) than that of MBP and PPP, and the IMP content of PPP was the lowest. Regarding the GMP content, except for the GMP content of TBP, which was significantly higher (*p* < 0.05) than that of WP, the GMP content of the other three breeds of pork showed similar significant differences (*p* < 0.05) to those of IMP. To evaluate the comprehensive umami taste of the five breeds of pork, the EUC values were calculated (Table 8). The data indicated that the EUC values of the five breeds of pork were significantly different (*p* < 0.05). The order of EUC values from large to small was WP > YNP > TBP > MBP > PPP. Therefore, the umami taste of WP was superior to that of the other breeds of pork.

As a pig is slaughtered, its carcass undergoes stiffness, aging, autolysis, and deterioration. Each process includes complicated biochemical reactions [70]. During the aging process, the metabolism of muscle cells and endogenous enzymes result in many nucleotides, which contribute to the pleasant taste of the pork as it is cooked [71]. The endogenous enzymes that participate in the production of umami-enhancing nucleotides are GMP synthase and AMP deaminase. GMP is mainly transferred from XMP under the catalysis of GMP synthase, IMP is mainly generated by AMP under the catalysis of AMP deaminase (EC: 3.5.4.6), and IMP can be decomposed into XMP and hypoxanthine (Hx) [71,72]. The umami taste of Glu can be enhanced using GMP and IMP because of their synergistic effect [65]. Therefore, the difference in pig breed, the effect of endogenous enzymes, and the synergistic enhancing effect of nucleotides and amino acids may explain the differences in umami taste between the five breeds of pork.

### 3.4. Volatile Aroma Compounds

Amino acids and nucleotides influence the taste of meat as they are chewed and consumed, but volatile aroma compounds are highly related to the smell of meat. To show the differences in smell, the volatile aroma compounds of the five breeds of pork were determined (Table 9). The data indicated that there were 11, 7, 6, 5, and 4 volatile aroma compounds in PPP, TBP, WP, YNP, and MBP, respectively. The odor activity value (OAV) indicated that there were five perceptible flavors in TBP, three in PPP and WP, and one in MBP. Acetaldehyde (pungent odor) is the characteristic perceptible flavor of WP, and linalool, 3-methyl-butanoic acid (pungent rancidity, fruity after dilution), and 3-hydroxy-2-butanone (creamy aroma) are the characteristic perceptible flavors of TBP. Nonanal (oil flavor) is a common perceptible flavor of TBP, PPP, and YNP. Moreover, 1-Octen-3-ol (peanuts and herbs) is the common perceptible flavor of TBP, PPP, and WP. The OAV of the five breeds of pork indicated that the perceptible flavor of TBP was greater than that of the other breeds of pork.

Volatile aroma compounds are generally produced through lipid oxidation, cooking processes [73], and microbial decomposition [74]. Fresh meat was used in the present study. Therefore, an explanation for the difference in the volatile aroma compounds may be the difference in pig breeds. This speculation could be supported by the application of volatile aroma compounds to distinguish pork breeds [75,76]. To control costs or meet other purposes, water-injected pork or artificial meat is available on the market [77,78]. The high similarity in color and texture between water-injected pork or artificial meat and real meat makes it difficult to be distinguished [76]. The characteristic volatile aroma compounds of PPP, TBP, WP, YNP, and MBP may be useful for determining the authenticity and freshness of pork.

### 3.5. Correlation Analysis

To demonstrate the relationship between the quality indices of the five breeds of pork, Pearson’s correlation was performed. The results in Table 10 indicated that there were highly significant correlations (*p* < 0.01) between moisture content and b * (0.961), EUC and meaty taste (0.976), SFA and MUFA (0.997), a * and umami amino acids (−0.984), SFA and sensory springiness (0.972), MUFA and sensory springiness (0.969), flavor and meaty taste (−0.962), and flavor and appearance (0.973).

The moisture content in the muscle surface can reflect light and, thus, influence color determination [55]. This may explain the high correlation between moisture content and b *. The EUC value is an index of umami taste; umami taste is also referred to as meaty taste [12]. The close relationship between umami and meaty tastes may result in a high correlation between the EUC value and meaty taste. Stearic acid had the third highest fatty acid content among the five breeds of pork (Table 3), and no other fatty acids showed strong correlations with pork texture, except for stearic acid, whose content was negatively correlated with the tenderness of pork [79]. Sensory springiness is a conversion index of tenderness. The relationship between fatty acids and pork texture may explain the high positive correlation between SFA and sensory springiness. The SFA content in pork was positively correlated with that of MUFA [80]. This may have resulted in a highly positive correlation between MUFA and sensory springiness. Retronasal aroma affects umami perception [81], and high-temperature treatment increases the redness and flavor of meat [82]. These results may explain why flavor and meaty taste were negatively correlated, and flavor and appearance were positively correlated.

## 4. Conclusions

The moisture content, sensory flavor, and overall acceptability of PPP were superior to those of the other breeds of pork. The fat content and EAA of YNP were significantly higher (*p* < 0.05) than those of the other samples. The protein content, EAA, and perceptible flavor of TBP were higher than those of the other pork samples. The protein nutrition indices suggested that the protein nutrition profiles of MBP and TBP were better than those of the other samples. The EUC value of WP was significantly higher (*p* < 0.05) than that of the other samples; however, the health risk of its fatty acids was higher than that of the other samples. The protein expression pattern analysis indicated that there were unique proteins (molecular weight of 113.257 kDa) in YPN, TBP (molecular weight of 15.948 kDa), and PPP (molecular weight of 15.948 kDa). The unique proteins and characteristic perceptible chemicals of PPP, TBP, WP, YNP, and MBP may be useful for distinguishing the authenticity and freshness of pork.

## Figures and Tables

**Table 1:** The scoring criteria of sensory parameters used in the sensory evaluations of the pork samples.

Items	Definition
Flavor	Overall meat aroma intensity of boiled pork
Meaty (Umami)	Meaty taste intensity
Sensory springiness	Restitution ability after pressing
Appearance	Red color intensity

**Table 2:** Chemical compositions of pork samples (mg/100 g).

Content Parameter	MBP	TBP	PPP	YNP	WP
Water	70.63 ± 0.19 c	71.07 ± 0.07 c	76.08 ± 0.01 a	70.98 ± 0.12 c	73.33 ± 0.05 b
Fat	3.1 ± 0.04 c	1.82 ± 0.03 e	2.58 ± 0.01 d	7.43 ± 0.1 a	4.15 ± 0.03 b
Protein	23.91 ± 0.29 b	25.12 ± 0.04 a	23.74 ± 0.31 b	21.93 ± 0.13 c	21.93 ± 0.57 c
Ash	1.16 ± 0.04 a	1.21 ± 0.07 a	1.12 ± 0.02 a	1.11 ± 0.08 a	1.24 ± 0.07 a

Means in the same row with different letters differ significantly (*p* < 0.05); MBP, manor black pigs; TBP, Tibetan black pigs; PPP, Pipa pigs; YNP, Yihao native pigs; and WP, white pigs.

**Table 3:** Fatty acid profiles of the five types of pork (mg/100 g).

Fatty Acids	MBP	TBP	PPP	YNP	WP
C10:0 (Decanoic acid)	1.1 ± 0.01 d	1.46 ± 0.04 c	3.31 ± 0.08 a	2.23 ± 0.07 b	1.24 ± 0.06 d
C12:0 (Lauric acid)	1.07 ± 0.01 cd	1.24 ± 0.02 c	2.25 ± 0.06 a	1.68 ± 0.07 b	1.05 ± 0.05 d
C14:0 (Myristic acid)	14.93 ± 0.43 c	16.43 ± 0.31 c	35.09 ± 1.05 a	23.56 ± 0.99 b	15.87 ± 0.71 c
C15:0 (Pentadecanoic acid)	/	/	/	1.07 ± 0.02	/
C16:0 (Palmitic acid)	262.11 ± 7.05 cd	296.53 ± 0.89 c	567.97 ± 20.39 a	373.04 ± 9.11 b	238.58 ± 10.18 d
C16:1n7 (palmitoleic acid)	24.55 ± 0.52 d	29.35 ± 0.26 d	79.66 ± 2.42 a	47.09 ± 1.93 b	34.96 ± 1.4 c
C17:0 (heptadecanoic acid)	2.09 ± 0.08 a	/	/	2.31 ± 0.06 a	/
C18:0 (stearic acid)	138.03 ± 5.58 c	149.79 ± 4.73 c	267.92 ± 11.20 a	179.1 ± 2.71 b	113.6 ± 4.22 d
C18:1 n9c (Oleic acid)	380.01 ± 8.93 d	486.66 ± 2.71 c	970.07 ± 37.47 a	574.7 ± 15.04 b	320.98 ± 12.17 d
C18:2 n6c (linoleic acid)	187.96 ± 3.25 a	190.65 ± 0.72 a	154.27 ± 3.55 b	185.74 ± 6.02 a	95.42 ± 3.45 c
C18:3 n3 (α-Linolenic acid)	8.98 ± 0.12 b	9.98 ± 0.11 a	4.51 ± 0.09 d	7.39 ± 0.34 c	/
C20:0 (arachidic acid)	/	/	4.59 ± 0.21 a	2.72 ± 0.01 b	/
C20:1 (cis-11-Eicosenoic acid)	5.56 ± 0.18 c	9.74 ± 0.31 b	13.72 ± 0.62 a	10.29 ± 0.24 b	3.56 ± 0.09 d
C20:2 (cis-11,14-Eicosadienoic Acid)	6.75 ± 0.19 b	8.52 ± 0.22 a	5.8 ± 0.17 c	7.21 ± 0.25 b	/
C20:3 n6 (8,11,14-Eicosatrienoicacid)	3.69 ± 0.08 ab	3.44 ± 0.06 b	3.56 ± 0.17 ab	3.88 ± 0.11 a	2.79 ± 0.07 c
C20:4 n6 (Arachidonic acid)	32.43 ± 0.66 a	25.07 ± 0.48 c	28.89 ± 1.05 b	30.66 ± 0.54 ab	21.96 ± 0.78 d
SFA	419.32 ± 13.14 cd	465.44 ± 5.26 c	881.13 ± 32.98 a	585.71 ± 13.01 b	370.34 ± 15.23 d
MUFA	410.12 ± 9.62 d	525.74 ± 3.28 c	1063.45 ± 40.51 a	632.07 ± 17.21 b	359.5 ± 13.66 d
PUFA	239.8 ± 4.31 a	237.65 ± 1.37 a	197.02 ± 5.03 b	234.87 ± 7.26 a	120.16 ± 4.29 c
IA	0.5 ± 0.003 d	0.48 ± 0.003 e	0.57 ± 0.001 b	0.55 ± 0.000 c	0.63 ± 0.004 a
IT	1.21 ± 0.013 d	1.15 ± 0.008 e	1.36 ± 0.002 b	1.28 ± 0.009 c	1.53 ± 0.006 a
NV	0.49 ± 0.003 d	0.46 ± 0.002 e	0.54 ± 0.001 b	0.52 ± 0.001 c	0.61 ± 0.003 a
h/H	2.21 ± 0.013 b	2.29 ± 0.008 a	1.93 ± 0.002 d	2.02 ± 0.004 c	1.73 ± 0.010 e

Mean values in the same row with different letters differ significantly (*p* < 0.05); MBP, manor black pigs; TBP, Tibetan black pigs; PPP, Pipa pigs; YNP, Yihao native pigs; WP, white pigs; ”/”, not detected (detection limit, 0.05%); SFA, saturated fatty acids; MUFA, monounsaturated fatty acids; PUFA, polyunsaturated fatty acids; IA, index of atherogenicity; FA, index of thrombogenicity; h/H, hypocholesterolemic/hypercholesterolemic ratio; IT, index of thrombogenicity; NV, nutritional value ratio.

**Table 4:** Content of hydrolyzed amino acids of pork (g/100 g).

Amino Acids	MBP	TBP	PPP	YNP	WP
Aspartic acid (Asp)	2.24 ± 0.01 b	2.33 ± 0.00 a	2.18 ± 0.01 c	2.00 ± 0.00 e	2.10 ± 0.00 d
Glutamate (Glu)	3.53 ± 0.01 b	3.74 ± 0.01 a	3.53 ± 0.02 b	3.25 ± 0.01 d	3.39 ± 0.00 c
Serine (Ser)	0.92 ± 0.00 ab	0.94 ± 0.00 a	0.90 ± 0.01 b	0.8 ± 0.02 d	0.86 ± 0.00 c
Glycine (Gly)	1.00 ± 0.00 b	1.04 ± 0.01 b	1.12 ± 0.01 a	0.91 ± 0.03 c	0.92 ± 0.01 c
Histidine (His)	1.22 ± 0.00 a	1.16 ± 0.00 b	1.08 ± 0.00 c	0.98 ± 0.01 d	0.98 ± 0.00 d
Arginine (Arg)	1.49 ± 0.01 b	1.57 ± 0.01 a	1.48 ± 0.01 b	1.35 ± 0.01 d	1.40 ± 0.01 c
Alanine (Met)	1.27 ± 0.00 b	1.32 ± 0.01 a	1.30 ± 0.01 ab	1.16 ± 0.01 d	1.20 ± 0.00 c
Proline (Pro)	0.73 ± 0.01 b	0.74 ± 0.01 b	0.79 ± 0.01 a	0.67 ± 0.01 c	0.68 ± 0.00 c
Threonine (Thr)	1.06 ± 0.01 b	1.10 ± 0.00 a	1.03 ± 0.00 c	0.95 ± 0.01 e	1.00 ± 0.00 d
Tyrosine (Tyr)	0.90 ± 0.01 b	0.94 ± 0.00 a	0.86 ± 0.01 c	0.83 ± 0.01 d	0.85 ± 0.00 cd
Valine (Val)	1.11 ± 0.01 ab	1.15 ± 0.01 a	1.06 ± 0.01 bc	1.01 ± 0.03 c	1.03 ± 0.00 c
Methionine (Met)	0.65 ± 0.01 ab	0.68 ± 0.02 a	0.65 ± 0.01 ab	0.59 ± 0 c	0.62 ± 0.00 b
Isoleucine (Ile)	1.07 ± 0.01 b	1.13 ± 0.01 a	1.04 ± 0.01 bc	0.99 ± 0.02 d	1.01 ± 0.00 cd
Leucine (Leu)	1.90 ± 0.01 b	1.98 ± 0.01 a	1.85 ± 0.01 c	1.73 ± 0.01 e	1.79 ± 0.00 d
Phenylalanine (Phe)	0.96 ± 0.00 b	1.00 ± 0.01 a	0.93 ± 0.01 c	0.88 ± 0 e	0.90 ± 0.01 d
Lysine (Lys)	2.05 ± 0.01 b	2.16 ± 0.03 a	1.99 ± 0.01 c	1.87 ± 0.02 e	1.93 ± 0.00 d
Total	22.1 ± 0.07 b	22.97 ± 0.12 a	21.8 ± 0.10 c	19.97 ± 0.02 e	20.65 ± 0.02 d
Content of EAA	39.820	40.050	39.270	40.160	40.100
AAS (Child)	1.583	1.564	1.550	1.547	1.6290
AAS (Adult)	3.189	2.886	2.843	2.793	2.793
EAAI (Child)	1.932	1.899	1.869	1.175	1.21
EAAI (Adult)	3.602	3.541	3.486	2.191	2.255
PER	31.657	31.388	31.107	31.373	32.519

Mean values in the same row with different letters differ significantly (*p* < 0.05); MBP, manor black pigs; TBP, Tibetan black pigs; PPP, Pipa pigs; YNP, Yihao native pigs; WP, white pigs; EAA, total essential amino acids; AAS, amino acid score; EAAI, essential amino acid index; PER, protein efficiency ratio [30].

**Table 5:** Texture profiles of the five types of pork.

Pork	Hardness (g)	Adhesiveness	Springiness	Cohesiveness (g.mm)	Gumminess	Chewiness	Resilience
MBP	11,111.23 ± 760.43 a	−2.96 ± 1.56 ab	0.59 ± 0.04 bc	0.58 ± 0.04 ab	6457.07 ± 341.60 a	3822.63 ± 339.81 a	0.22 ± 0.03 ab
TBP	8513.72 ± 264.68 c	−4.82 ± 0.00 b	0.65 ± 0.03 b	0.57 ± 0.01 b	4772.56 ± 272.22 c	2788.32 ± 156.21 b	0.19 ± 0.01 b
PPP	5757.24 ± 141.86 d	−49.25 ± 2.57 a	0.78 ± 0.04 a	0.63 ± 0.02 a	3608.13 ± 201.18 d	2836.21 ± 321.34 b	0.23 ± 0.02 a
YNP	9616.89 ± 430.94 b	−1.50 ± 0.36 c	0.62 ± 0.03 b	0.58 ± 0.04 ab	5747.42 ± 84.78 b	3470.91 ± 312.26 a	0.20 ± 0.02 ab
WP	9135.96 ± 183.80 bc	−1.11 ± 0.19 c	0.54 ± 0.03 c	0.56 ± 0.02 b	5109.63 ± 242.36 c	2769.87 ± 111.65 b	0.18 ± 0.01 b

Mean values in the same row with different letters differ significantly (*p* < 0.05); MBP, manor black pigs; TBP, Tibetan black pigs; PPP, Pipa pigs; YNP, Yihao native pigs; and WP, white pigs.

**Table 6:** Sensory evaluations of the five types of pork.

Parameter	MBP	TBP	PPP	YNP	WP
Flavor	14.66 ± 1.88 a	14.66 ± 0.67 a	15.33 ± 1.68 a	14.33 ± 2.17 a	14.33 ± 0.66 a
Meaty taste	14.43 ± 1.00 a	14.69 ± 0.85 a	14.00 ± 2.57 a	15.00 ± 0.57 a	14.88 ± 1.33 a
Springiness	14.33 ± 1.20 a	14.33 ± 0.88 a	16.00 ± 1.37 a	14.66 ± 1.33 a	14.33 ± 2.19 a
Appearance	15.60 ± 1.33 a	15.66 ± 2.16 a	16.72 ± 1.73 a	14.66 ± 2.51 a	15.11 ± 2.57 a

Means in the same row with different letters differ significantly (*p* < 0.05); MBP, manor black pigs; TBP, Tibetan black pigs; PPP, Pipa pigs; YNP, Yihao native pigs; and WP, white pigs.

**Table 7:** The volatile aroma compounds of the five types of pork (μg/kg).

RT/min	CAS	Name	Formula	Concentration (μg/kg)					Threshold (μg/kg)	OAV				
				MBP	TBP	PPP	YNP	WP		MBP	TBP	PPP	YNP	WP
4.564	66-25-1	Hexanal	C_6_H_12_O	3.22 ± 0.02	/	13.00 ± 0.01	13.57 ± 0.05	6.41 ± 0.01	5.00	0.64	/	2.60	2.71	1.28
14.575	124-19-6	Nonanal	C_9_H_18_O	/	3.36 ± 0.01	9.70 ± 0.00	4.42 ± 0.02	/	1.10	/	3.05	8.82	4.02	/
18.492	75-07-0	Acetaldehyde	C_2_H_4_O	/	/	/	/	0.42 ± 0.00	25.10	/	/	/	/	0.02
9.866	372-66-7	6-amino-2-methyl-2-Heptanol	C_8_H_19_NO	/	/	0.55 ± 0.00	0.19 ± 0.00	/	/	/	/	/	/	/
10.104	3391-86-4	1-Octen-3-ol	C_8_H_16_O	/	2.40 ± 0.01	5.04 ± 0.00	/	19.75 ± 0.04	1.50	/	1.60	3.36	/	13.17
14.435	78-70-6	Linalool	C_10_H_18_O	/	5.06 ± 0.01	/	/	/	0.22	/	23.00	/	/	/
6.007	503-74-2	3-Methyl-butanoic acid	C_5_H_10_O_2_	/	15.08 ± 0.04	/	/	/	70.00	/	0.22	/	/	/
28.961	166273-38-7	Pentanoic acid, 5-hydroxy-, 2,4-di-t-butylphenyl esters	C_19_H_30_O_3_	/	/	3.02 ± 0.00	2.91 ± 0.01	/	/	/	/	/	/	/
6.835	2439-54-5	N-Methyl-1-octanamine	C_9_H_21_N	/	/	1.04 ± 0.00	/	/	/	/	/	/	/	/
8.041	4025-37-0	2-(aziridin-1-yl) ethanamine	C_4_H_10_N_2_	/	/	1.16 ± 0.00	/	/	/	/	/	/	/	/
18.498	60-35-5	Acetamide	C_2_H_5_NO	0.23 ± 0.00	/	/	/	0.45 ± 0.00	/	/	/	/	/	/
3.256	513-86-0	3-Hydroxy-2-Butanone; Acetoin	C_4_H_8_O_2_	/	22.41 ± 0.06	/	/	/	14.00	/	1.60	/	/	/
10.309	13475-82-6	2,2,4,6,6-Pentamethyl-heptane	C_12_H_26_	3.57 ± 0.02	17.63 ± 0.04	20.56 ± 0.01	16.59 ± 0.07	/	/	/	/	/	/	/
17.927	563-16-6	3,3-Dimethyl-hexane	C_8_H_18_	/	/	2.52 ± 0.00	/	/	/	/	/	/	/	/
17.921	4418-61-5	5-Aminotetrazole	CH_3_N_5_	/	/	/	/	3.59 ± 0.01	/	/	/	/	/	/
19.824	1014-60-4	1,3-Ditert-butyl-benzene	C_14_H_22_	/	/	1.77 ± 0.00	/	/	/	/	/	/	/	/
33.11	1111-78-0	Ammonium carbamate	CH_6_N_2_O_2_	0.13 ± 0.00	0.15 ± 0.00	1.77 ± 0.00	/	0.52 ± 0.00	/	/	/	/	/	/

MBP, manor black pigs; TBP, Tibetan black pigs; PPP, Pipa pigs; YNP, Yihao native pigs; and WP, white pigs.

**Table 8:** Contents of free amino acids of pork (mg/100 g).

Amino Acids	MBP	TBP	PPP	YNP	WP
Asp	11.93 ± 0.12 a	12.66 ± 0.55 a	6.81 ± 0.12 b	12.71 ± 0.56 a	7.42 ± 0.295 b
Thr	25.95 ± 0.97 a	18.48 ± 0.32 b	15.57 ± 0.46 c	14.95 ± 0.61 c	13.47 ± 0.40 c
Ser	23.67 ± 0.57 a	16.73 ± 0.36 b	15.15 ± 0.20 cd	16.42 ± 0.39 bc	13.98 ± 0.06 d
Glu	12.53 ± 0.11 d	17.43 ± 0.09 b	9.04 ± 0.035 e	15.79 ± 0.66 c	20.87 ± 0.23 a
Gly	11.10 ± 0.39 a	9.68 ± 0.26 c	10.16 ± 0.19 cb	8.24 ± 0.11 d	10.72 ± 0.02 ab
Ala	37.77 ± 0.29 a	26.15 ± 1.05 c	18.75 ± 0.29 d	30.92 ± 0.59 b	16.29 ± 0.34 e
Val	16.34 ± 0.46 a	15.93 ± 0.23 a	10.11 ± 0.18 c	13.08 ± 0.3 b	3.08 ± 0.11 d
Met	5.98 ± 0.03 c	4.41 ± 0.10 d	6.46 ± 0.05 b	6.13 ± 0.18 bc	7.12 ± 0.01 a
Ile	10.41 ± 0.35 a	5.63 ± 0.14 d	7.77 ± 0.24 b	4.95 ± 0.03 d	6.80 ± 0.14 c
Leu	11.46 ± 0.22 a	9.72 ± 0.15 b	5.88 ± 0.31 c	3.86 ± 0.16 d	6.51 ± 0.11 c
Tyr	7.99 ± 0.01 c	10.11 ± 0.37 a	6.85 ± 0.10 d	7.21 ± 0.03 d	9.21 ± 0.17 b
Phe	10.9 ± 0.23 b	10.12 ± 0.33 c	12.14 ± 0.16 a	6.78 ± 0.14 d	4.73 ± 0.13 e
Lys	15.47 ± 0.18 a	8.70 ± 0.05 c	5.41 ± 0.15 e	9.35 ± 0.02 b	6.63 ± 0.23 d
His	505.18 ± 6.70 a	446.59 ± 1.08 b	403.64 ± 6.35c	344.81 ± 4.08d	344.94 ± 3.57 d
Arg	10.17 ± 0.45 a	7.76 ± 0.34 b	7.59 ± 0.04 b	3.96 ± 0.01 c	7.97 ± 0.23 b
Pro	10.90 ± 0.15 c	13.65 ± 0.32 b	8.77 ± 0.25 d	14.83 ± 0.69 b	19.73 ± 0.40 a
Umami amino acids	24.45 ± 0.22 c	30.09 ± 0.46 a	15.84 ± 0.08 d	28.50 ± 0.10 b	28.28 ± 0.52 b
Sweet amino acids	109.38 ± 1.21 a	84.67 ± 0.43 b	68.39 ± 0.32 d	85.35 ± 1.39 b	74.18 ± 1.21 c
Bitter amino acids	570.43 ± 8.43 a	500.13 ± 1.90 b	453.58 ± 6.47 c	383.55 ± 3.32 d	381.13 ± 4.29 d
Total amount of AA	727.68 ± 10.03 a	633.70 ± 1.45 b	550.07 ± 6.91 c	513.94 ± 1.81 d	499.39 ± 3.49 d

Mean values in the same row with different letters differ significantly (*p* < 0.05); umami amino acids are the sum of Glu and Asp; sweet amino acids are the sum of Thr, Ser, Gly, Ala, and Pro; bitter amino acids are the sum of Val, Met, Ile, Leu, Phe, His, and Arg; MBP, manor black pigs; TBP, Tibetan black pigs; PPP, Pipa pigs; YNP, Yihao native pigs; and WP, white pigs.

**Table 9:** Contents of nucleotides in the five types of pork (mg/100 g).

Nucleotides	MBP	TBP	PPP	YNP	WP
5′-IMP	293.9 ± 0.28 d	298.66 ± 0.05 c	263.54 ± 0.02 e	367.85 ± 0.43 a	313.97 ± 0.58 b
5′-GMP	2.57 ± 0.00 d	3.17 ± 0.00 b	2.15 ± 0.00 e	3.45 ± 0.01 a	3.05 ± 0.02 c
5′-UMP	4.03 ± 0.09 d	8.76 ± 0.04 b	4.37 ± 0.00 c	3.29 ± 0.00 e	9.55 ± 0.04 a
5′-CMP	1.81 ± 0.05 a	1.83 ± 0.00 a	1.09 ± 0.00 c	1.2 ± 0.04 b	1.85 ± 0.00 a
5′-XMP	6.56 ± 0.01 b	6.28 ± 0.01 c	2.45 ± 0.00 e	14.59 ± 0 a	2.68 ± 0.01 d
5′-AMP	6.09 ± 0.00 c	4.31 ± 0.00 d	2.59 ± 0.00 e	11.78 ± 0.01 a	6.62 ± 0.00 b
Glu	12.53 ± 0.11 d	17.43 ± 0.09 b	9.04 ± 0.035 e	15.79 ± 0.66 c	20.87 ± 0.23 a
ASP	11.93 ± 0.12 a	12.66 ± 0.55 a	6.81 ± 0.12 b	12.71 ± 0.56 a	7.42 ± 0.295 b
EUC	4.99 ± 0.05 d	6.96 ± 0.02 c	3.15 ± 0.01 e	7.9 ± 0.28 b	8.45 ± 0.11 a

Mean values in the same row with different letters differ significantly (*p* < 0.05); MBP, manor black pigs; TBP, Tibetan black pigs; PPP, Pipa pigs; YNP, Yihao native pigs; and WP, white pigs.

**Table 10:** Correlation analysis of the five types of pork.

Indices	Water Content	Protein Content	Fat Content	EAAI	PER	SFA	MUFA	PUFA	a *	b *	L *	EUC	Umami Amino Acids	Sweet Amino Acids	Bitter Amino Acids	Flavor	Meaty Taste	Sensory Springiness	Appearance
Water content	1	−0.088	−0.28	0.086	−0.09	0.711	0.719	−0.537	0.74	0.961 **	0.597	−0.535	−0.797	−0.768	−0.295	0.708	−0.653	0.845	0.705
Protein content	−0.088	1	−0.828	0.923 *	−0.504	0.097	0.154	0.54	0.014	0.102	0.055	−0.505	−0.14	0.247	0.789	0.532	−0.516	0.058	0.604
Fat content	−0.28	−0.828	1	−0.832	0.1	−0.043	−0.103	−0.001	−0.173	−0.445	−0.519	0.52	0.304	0.01	−0.644	−0.596	0.636	−0.137	−0.751
EAAI	0.086	0.923 *	−0.832	1	−0.52	0.239	0.275	0.498	0.35	0.327	−0.017	−0.764	−0.44	0.313	0.883 *	0.708	−0.76	0.245	0.765
PER	−0.09	−0.504	0.1	−0.52	1	−0.731	−0.739	−0.771	−0.394	−0.181	0.468	0.618	0.448	−0.01	−0.309	−0.628	0.499	−0.58	−0.477
SFA	0.711	0.097	−0.043	0.239	−0.731	1	0.997 **	0.153	0.772	0.713	−0.024	−0.701	−0.817	−0.523	−0.13	0.807	−0.675	0.972 **	0.665
MUFA	0.719	0.154	−0.103	0.275	−0.739	0.997 **	1	0.151	0.744	0.722	0.026	−0.701	−0.804	−0.55	−0.115	0.827	−0.686	0.969 **	0.696
PUFA	−0.537	0.54	−0.001	0.498	−0.771	0.153	0.151	1	−0.04	−0.392	−0.785	−0.298	0.038	0.593	0.586	0.153	−0.123	−0.036	0.043
a *	0.74	0.014	−0.173	0.35	−0.394	0.772	0.744	−0.04	1	0.842	−0.017	−0.866	−0.984 **	−0.203	0.147	0.805	−0.853	0.856	0.73
b *	0.961 **	0.102	−0.445	0.327	−0.181	0.713	0.722	−0.392	0.842	1	0.512	−0.727	−0.900 *	−0.58	−0.026	0.837	−0.828	0.853	0.848
L *	0.597	0.055	−0.519	−0.017	0.468	−0.024	0.026	−0.785	−0.017	0.512	1	0.073	−0.09	−0.685	−0.283	0.187	−0.145	0.138	0.346
EUC	−0.535	−0.505	0.52	−0.764	0.618	−0.701	−0.701	−0.298	−0.866	−0.727	0.073	1	0.904 *	−0.018	−0.552	−0.934 *	0.976 **	−0.742	−0.895 *
Umami amino acids	−0.797	−0.14	0.304	−0.44	0.448	−0.817	−0.804	0.038	−0.984 **	−0.900 *	−0.09	0.904 *	1	0.288	−0.173	−0.893 *	0.913 *	−0.901 *	−0.833
Sweet amino acids	−0.768	0.247	0.01	0.313	−0.01	−0.523	−0.55	0.593	−0.203	−0.58	−0.685	−0.018	0.288	1	0.714	−0.299	0.116	−0.581	−0.286
Bitter amino acids	−0.295	0.789	−0.644	0.883 *	−0.309	−0.13	−0.115	0.586	0.147	−0.026	−0.283	−0.552	−0.173	0.714	1	0.358	−0.499	−0.135	0.425
Flavor	0.708	0.532	−0.596	0.708	−0.628	0.807	0.827	0.153	0.805	0.837	0.187	−0.934 *	−0.893 *	−0.299	0.358	1	−0.962 **	0.851	0.973 **
Meaty taste	−0.653	−0.516	0.636	−0.76	0.499	−0.675	−0.686	−0.123	−0.853	−0.828	−0.145	0.976 **	0.913 *	0.116	−0.499	−0.962 **	1	−0.754	−0.962 **
Springiness	0.845	0.058	−0.137	0.245	−0.58	0.972 * *	0.969 **	−0.036	0.856	0.853	0.138	−0.742	−0.901 *	−0.581	−0.135	0.851	−0.754	1	0.745
Appearance	0.705	0.604	−0.751	0.765	−0.477	0.665	0.696	0.043	0.73	0.848	0.346	−0.895 *	−0.833	−0.286	0.425	0.973 **	−0.962 **	0.745	1

“**” Represents a significant correlation at the 0.01 level; “*” represents a significant correlation at the 0.05 level; EAAI, essential amino acid index; PER, protein efficiency ratio; SFA, saturated fatty acids; MUFA, monounsaturated fatty acids; PUFA, polyunsaturated fatty acids; EUC, and equivalent umami calculation.

## Data Availability

All related data and methods are presented in this paper. Additional inquiries should be addressed to the corresponding author.
